# One-stage operation for thoracic aortic arch aneurysm and left lung carcinoma: a case report

**DOI:** 10.1186/s13019-016-0440-0

**Published:** 2016-04-11

**Authors:** Takashi Makino, Kota Kawada, Hiroshi Masuhara, Yoshinobu Hata, Hajime Otsuka, Satoshi Koezuka, Naobumi Tochigi, Kazutoshi Shibuya, Yoshinori Watanabe, Akira Iyoda

**Affiliations:** Division of Chest Surgery, Toho University School of Medicine, Tokyo, Japan; Division of Cardiovascular Surgery, Toho University School of Medicine, Tokyo, Japan; Department of Surgical Pathology, Toho University School of Medicine, Tokyo, Japan

**Keywords:** Surgery, One-stage operation, Aortic arch aneurysm, Lung carcinoma

## Abstract

**Background:**

The simultaneous surgical treatment of thoracic aortic arch aneurysm (TAA) and lung carcinoma is extremely rare.

**Case presentation:**

We report the simultaneous surgical treatment of TAA and squamous cell carcinoma of the lung in a 72-year-old Japanese man. We performed a one-stage operation that consisted of aortic arch replacement for aortic arch aneurysm with a 3-branched artificial vessel under separate cerebral and systemic extracorporeal circulation, and left upper lobectomy for lung cancer via a left lateral thoracotomy.

**Conclusions:**

Although patients should be carefully selected for this procedure, the simultaneous surgical treatment of TAA and lung carcinoma can be performed safely.

## Background

The surgical management of coexisting cardiovascular disease and lung cancer remains controversial [1, 2]. The treatment of patients with resectable lung carcinoma and coexisting cardiac disease is problematic because of the increased operative mortality of lung resection [3]. Usually, the surgical methods are staged, with cardiac surgery performed first followed by lung resection at a later date. However, when resection for lung carcinoma is delayed, the immunosuppressive effects of cardiopulmonary bypass may have a harmful effect on the growth of lung carcinoma, leading to metastases [3]. In this report, we present a patient who had a one-stage operation that consisted of aortic arch replacement for aortic arch aneurysm with a 3-branched artificial vessel under separate cerebral and systemic extracorporeal circulation, and left upper lobectomy for lung carcinoma. The management of the patient with TAA and lung cancer is challenging, and previous reports on the simultaneous surgical treatment of TAA and lung carcinoma are extremely rare.

## Case presentation

**Fig. 1:**
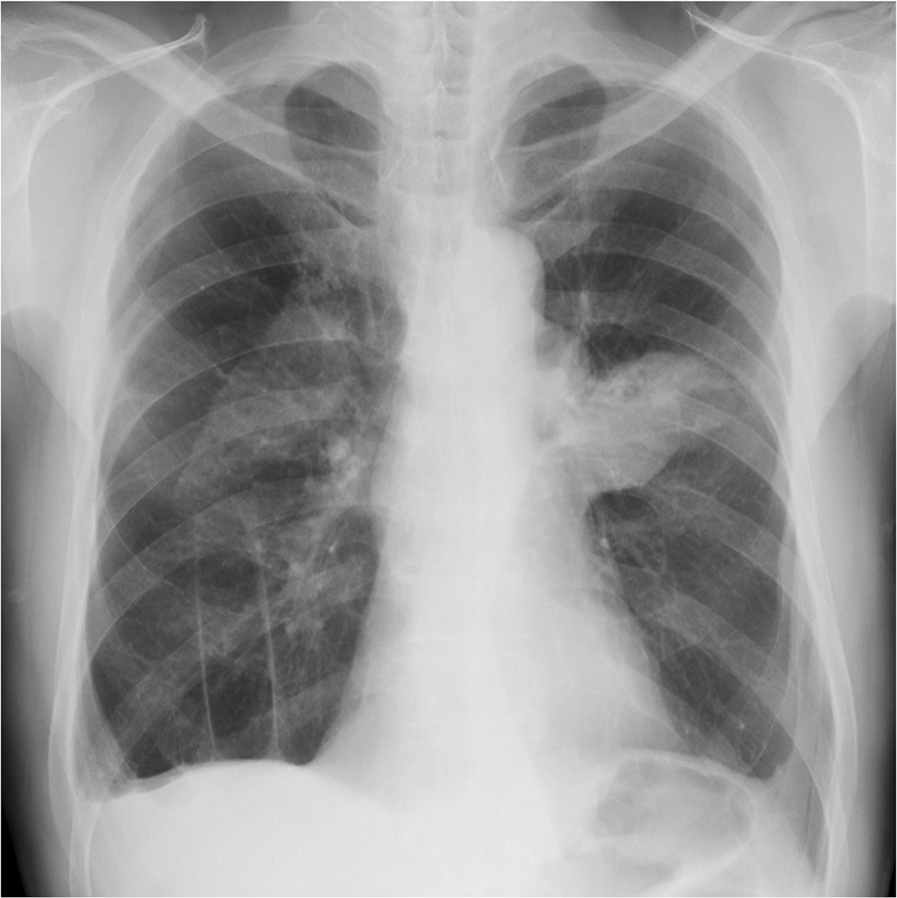
A chest X-ray revealed a mass shadow in the left lung field and a positive silhouette sign for the left second arch

**Fig. 2:**
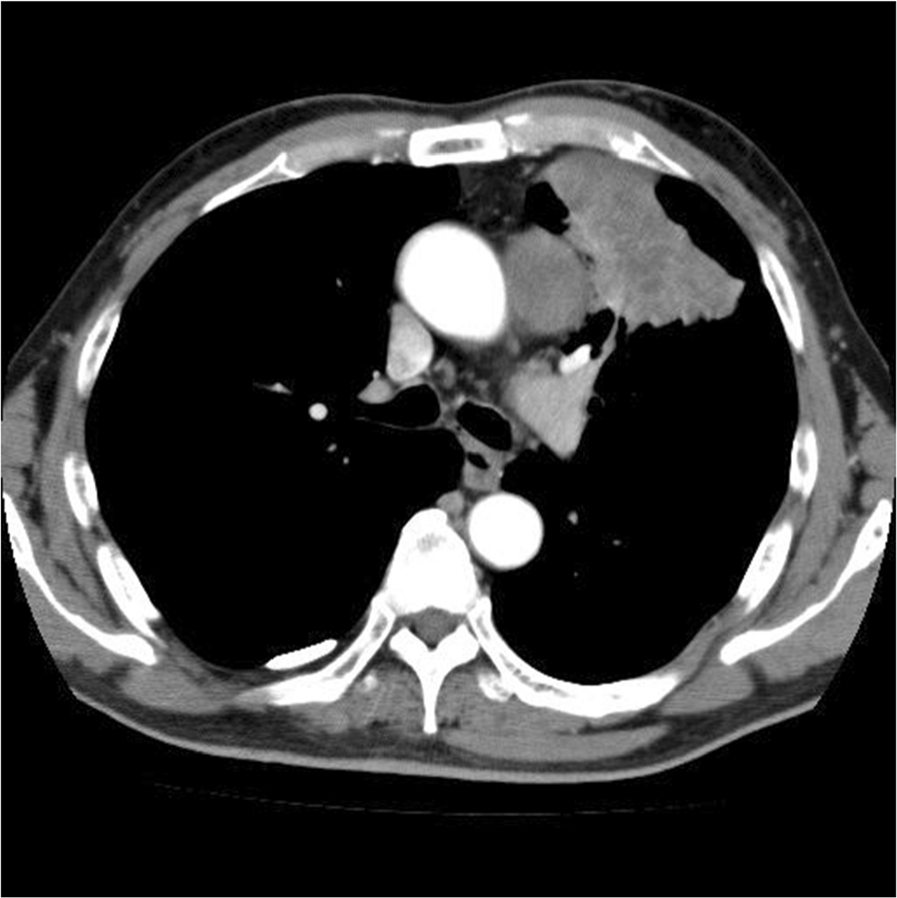
Enhanced computed tomography of the chest showed a 60-mm distal aortic arch aneurysm and a 60-mm mass shadow in the left upper lobe. The aortic arch aneurysm was connected to the pulmonary mass in the left upper lobe

**Fig. 3:**
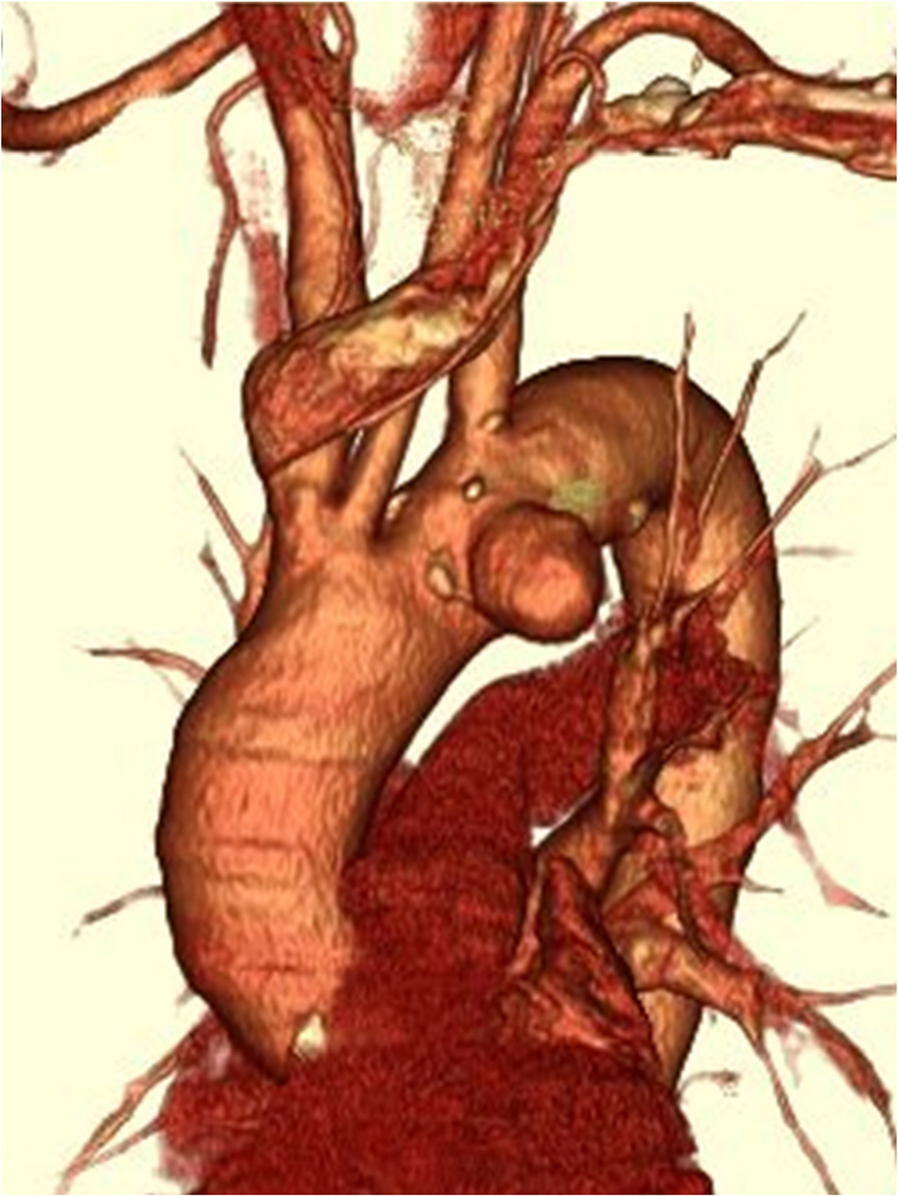
Three-dimensional computed tomography revealed that the aortic aneurysm was located in front of the arch near the left subclavian artery, and the brachiocephalic artery and left common carotid artery branched from the aorta with a common duct

A 72-year-old man was referred to our hospital because of an abnormal pulmonary shadow found on a routine chest X-ray. Chest X-rays revealed a mass shadow in the left middle lung field and a positive silhouette sign for the left second arch (Fig. 1). The aortic arch aneurysm was saccular aneurysm associated with a greater risk of rupture, and connected to the pulmonary mass in the left upper lobe with a possible risk of tumor adhesion or invasion to the aneurysm (Fig. 2). Three-dimensional CT revealed that the aortic aneurysm was located in front of the arch near the left subclavian artery, and the brachiocephalic and left common carotid arteries branched from the aorta with a common duct (Fig. 3). These findings led to the diagnosis of aortic arch aneurysm and lung carcinoma. There were no distant metastases of the lung carcinoma (clinical stage T2bN0M0 stage IIA). We decided to perform left upper lobectomy and patch angioplasty if the arch of aorta between the left common carotid artery and the left subclavian artery could be temporarily occluded. If not, we planned to perform aortic arch replacement under selective cerebral perfusion with systemic extracorporeal circulation through the groin.

A fourth intercostal thoracotomy was performed. As the lung carcinoma adhered to the aortic arch aneurysm but did not invade it, we could detach the visceral pleura of the left upper lobe from the aortic arch aneurysm. We performed left upper lobectomy. After that, we confirmed that the aneurysm was proximal to the left subclavian artery. Because of the position of the TAA, it was difficult to perform patch angioplasty. We decided to perform aortic arch replacement for the aortic arch aneurysm with a 3-branched artificial vessel under selective cerebral perfusion with systemic extracorporeal circulation. The thoracotomy was extended to the right with a transverse incision of the sternum.

A 30-mm intimal defect was observed in the aorta, and half of the aneurysm was filled with organized thrombus. It was morphologically diagnosed as pseudoaneurysm. Under deep hypothermia, circulatory arrest and separate cerebral and systemic extracorporeal circulation, aortic arch replacement was performed with a 3-branched artificial vessel. First, the brachiocephalic artery and the left common carotid artery were rebuilt by the first branch of the graft. The second branch was ligated, and the subclavian artery was rebuilt by the third branch. Forming a stump, the distal aorta was anastomosed to the entire circumference of the graft and was reinforced with Teflon felt strips. The operative time was 683 min, the extracorporeal circulation time was 213 min, and the aortic cross-clamp time was 121 min. He was diagnosed with a moderately differentiated squamous cell carcinoma that was graded pathologically as T2bN0M0-stageIIA.

After the surgical treatment, postoperative pneumonia developed that was treated by ventilator management. The patient was removed from the ventilator on the 23rd postoperative day. He was discharged from our hospital on the 72nd postoperative day.

## Discussion

A one-stage operation involving graft replacement for TAA and resection of left lung carcinoma is extremely rare. There were no cases in the English literature where a one-stage operation was performed for aortic arch aneurysm and left lung carcinoma. The decision to perform simultaneous operations in the same operative field was made with consideration of the risk to the patient and the disease stage of both lesions. Early surgical intervention with a one-stage procedure was necessary in our case, since the patient had a 60-mm saccular aneurysm with a high risk of rupture, and the lung carcinoma was suspected to invade the aneurysm.

TAA can occur as either a descending aorta aneurysm or arch aortic aneurysm. When lung cancer coexists with a descending aortic aneurysm, a one-stage or two-stage operation can be performed without the need for extracorporeal circulation by placing a stent graft in the descending thoracic aorta [4–6]. However, when lung carcinoma coexists with an aortic arch aneurysm, it is necessary to perform a one-stage operation under cardiopulmonary bypass, although cardiopulmonary bypass has a substantial risk of postoperative bleeding, pulmonary dysfunction and tumor dissemination [1, 7]. Thoracic endovascular aortic repair (TEVAR) is an attractive alternative. There has been rapid technological development and widespread uptake of this approach for thoracic aortic disease, including hybrid grafting techniques to the aortic arch, although long-term outcomes and the relative advantages and disadvantages are still controversial [8]. Although operative complications must be carefully managed, we successfully performed left upper lobectomy and aortic arch replacement under separate but simultaneous cerebral and systemic extracorporeal circulation. Furthermore, we can safely perform a one-stage operation in patients with a high risk aneurysm rupture with the expectation of long-term survival after resection of the lung carcinoma.

## Conclusion

In the present case, a one-stage operation was successfully performed, which consisted of aortic arch replacement for aortic arch aneurysm with a 3-branched artificial vessel under separate cerebral and systemic extracorporeal circulation, and left upper lobectomy for lung cancer via a left lateral thoracotomy.

### Informed consent

Written informed consent was obtained from the patient for publication of this Case report and any accompanying images. A copy of the written consent is available for review by the Editor-in-Chief of this journal.
